# A Unique Presentation of Concurrent Duodenal and Peritoneal Metastasis From Head and Neck Cancer

**DOI:** 10.7759/cureus.12859

**Published:** 2021-01-22

**Authors:** Ahmed Ahmed, Christopher Lenza

**Affiliations:** 1 Internal Medicine, Rutgers University, Newark, USA; 2 Gastroenterology and Hepatology, Veterans Affairs Medical Center, East Orange, USA

**Keywords:** esophagogastroduodenoscopy, immunohistochemistry staining, duodenal metastasis, head and neck neoplasms, squamous cell neoplasm

## Abstract

Metastatic disease to the duodenum or peritoneum from a primary head and neck carcinoma is an extremely rare presentation. We report the case of a 68-year-old male with a history of head and neck squamous cell carcinoma (HNSCC) who presented with worsening nausea, abdominal pain, postprandial vomiting, and early satiety for over two months. Prior to this presentation, he was evaluated for several postauricular lumps, with computerized tomography (CT) scan showing a supraglottic mass and an excisional biopsy of a postauricular nodule confirming metastatic HNSCC. A CT scan of the chest, abdomen, and pelvis during the admission showed worsening lymphadenopathy in the mediastinum and hilar regions, as well as new ascites and peritoneal lesions. Esophagogastroduodenoscopy showed a large erythematous nodular lesion in the second portion of the duodenum occupying approximately one-third of the lumen circumference. Similar to the previously worked up nodule, histology from the duodenal mass biopsies showed metastatic poorly differentiated squamous cell carcinoma that was strongly positive for p63 and p16. Thus, we report the first case of concurrent duodenal and peritoneal metastasis from an HNSCC.

## Introduction

Head and neck squamous cell carcinoma (HNSCC) most commonly metastasize to the lung, followed by the bone and liver [1-3]. It is highly uncommon for HNSCC to metastasize to the gastrointestinal (GI) tract and small intestine. Only a few cases have been reported in the literature of HNSCC metastasizing specifically to the duodenum, further illustrating its rarity [4-9]. Moreover, only a handful of cases have reported peritoneal metastasis (PM) from an HNSCC [10-13]. Unfortunately, distant metastasis from HNSCC is associated with a poor prognosis, even after initial treatment [14]. Here, we report a case of a patient presenting with worsening abdominal pain, postprandial vomiting, and early satiety due to simultaneous HNSCC metastasis to the second portion of the duodenum and peritoneum. This article was previously presented as a meeting abstract at the 2019 American College of Gastroenterology Annual Scientific Meeting on October 29, 2019.

## Case presentation

A 68-year-old male with past medical history notable for squamous cell carcinoma (SCC) of the right aryepiglottic fold and vallecula (diagnosed three months prior to presentation), diabetes mellitus, gastroesophageal reflux disease, tobacco use, and chronic obstructive pulmonary disease presented with worsening nausea, postprandial, nonbloody, nonbilious vomiting, 10-pound weight loss, and early satiety over a two-month period. The patient also complained of worsening generalized, dull abdominal pain, especially after eating. He also complained of episodes of dark stools over the past month. He denied any dysphagia, odynophagia, hematochezia, hematuria, or dysuria. He also denied use of alcohol, nonsteroidal anti-inflammatory drugs, or anticoagulants. He reported a 30-pack-year history of tobacco use and denied any family history of cancer. Laboratory studies were significant for elevated blood urea nitrogen (29 mg/dL), elevated creatinine (2.2 mg/dL), normocytic anemia (Hgb of 10.1 g/dL), and electrolyte imbalance, including hypercalcemia (10.7 mg/dL) and hypokalemia (3.1 mmol/L). Physical examination was notable for cervical lymphadenopathy (LAD), upper abdominal diffuse tenderness, abdominal distension, tachycardia to 110 beats per minute, and mild bilateral lower extremity edema.

**Figure 1:**
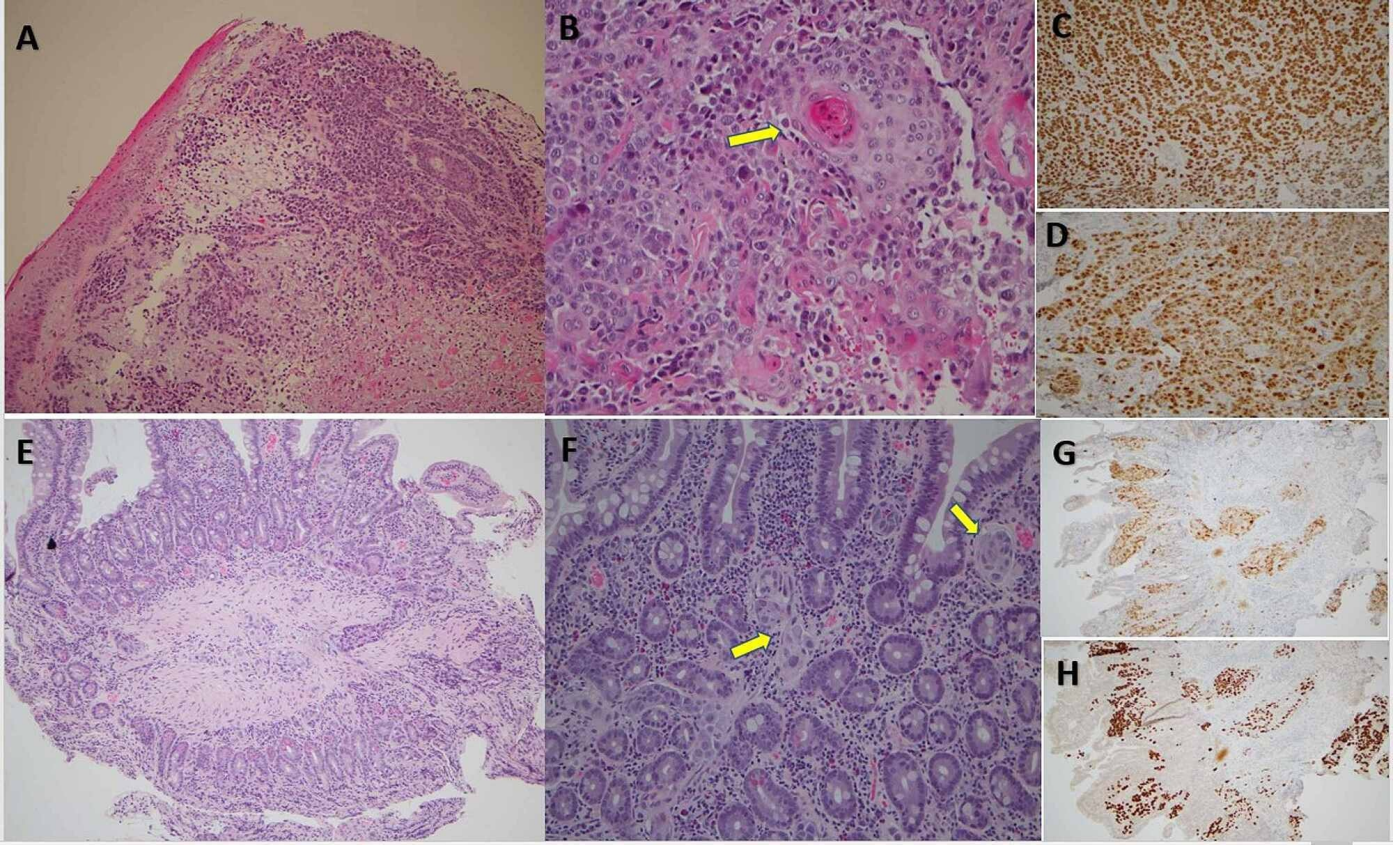
Histological and immunohistohemical stains of biopsies. Biopsy images from the postauricular nodule during the HNSCC workup (A-D) as well as the duodenal biopsy (E-H). (A and E) H&E stain (10×) showing poorly differentiated SCC. (B and F) H&E stain (20×) showing keratinization (arrow). (C) Diffusely positive P63 staining (20×). (D) Diffusely positive P16 (cytoplasmic and nuclear) staining (20×). (G) Areas of positive P63 staining (10×). (H) Areas of positive p16 staining (10×). H&E, hematoxylin and eosin; HNSCC, head and neck squamous cell carcinoma; SCC, squamous cell carcinoma

Three months prior to the current admission, the patient complained of enlarging left postauricular “lumps.” Computerized tomography (CT) scan of the neck and chest was done and showed bilateral cervical and mediastinal hilar LAD and a well-defined nodular lesion in the right aryepiglottic fold and vallecula with extension to the right false vocal cord measuring approximately 2 × 2 cm, which was concerning for malignancy. An excisional biopsy was done of the left postauricular nodule and was sent for cytology. The results revealed poorly differentiated SCC that was positive for p63 and p16 but negative for CK-20, CK-7, CTX-2, TTF-1, synaptophysin, prostate-specific antigen (PSA), and chromogranin (Figure 1). Consequently, the patient was scheduled for a positron emission topography scan and was scheduled for follow-up with oncology and radiation oncology for further workup and treatment of his newly diagnosed SCC. Unfortunately, the patient was hospitalized before he could see the specialists.

**Figure 2:**
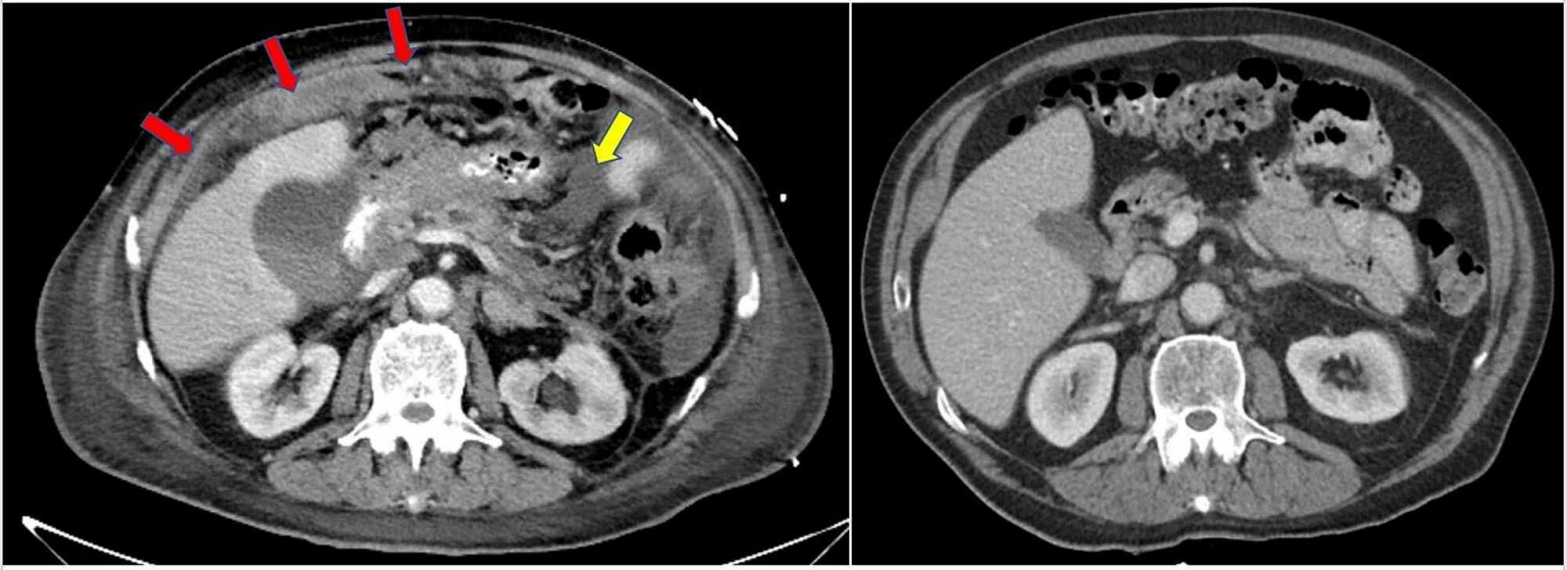
CT scan image of the abdomen (left) showing new omental carcinomatosis (red arrows) and ascites (yellow arrow) compared to the CT scan done three months prior (right). CT, computerized tomography

**Figure 3:**
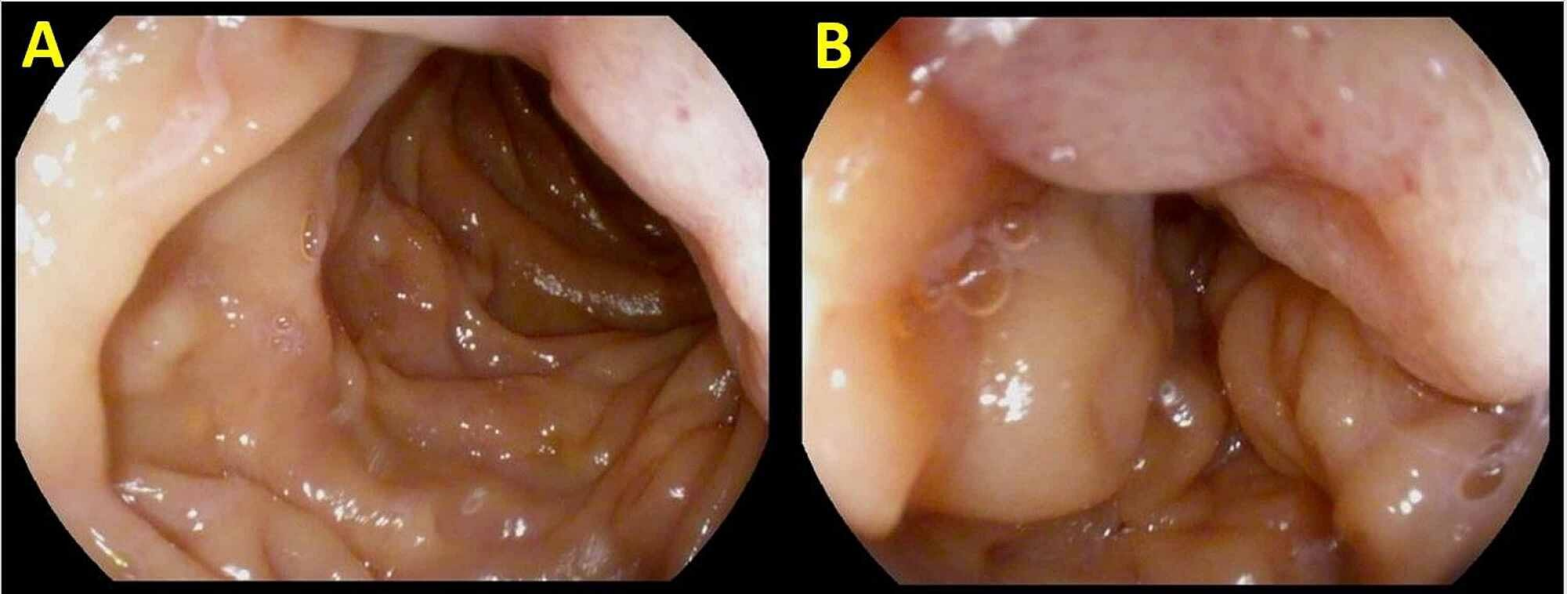
Esophagogastroduodenoscopy images. Nodular, erythematous, friable lesion of the duodenum (A) during EGD later found to be a metastatic lesion from the primary HNSCC. A close-up view of the metastatic lesion (B) illustrates how it occupies approximately one-third of the circumference of the lumen and a distinct change in appearance can be appreciated with the lesion being more erythematous, friable, and containing punctate hemorrhages compared to the surrounding mucosa. EGD, esophagogastroduodenoscopy; HNSCC, head and neck squamous cell carcinoma

During the hospital admission, he underwent a CT scan of the chest, abdomen, and pelvis and was found to have new peritoneal carcinomatosis and ascites, worsening hilar and mediastinal LAD, new common bile duct dilation and pancreatic duct dilatation, and new mesenteric LAD (Figure 2). An esophagogastroduodenoscopy (EGD) was done in light of the patient’s history of dark stools, anemia, presenting symptoms, and CT findings. A large erythematous nodular lesion was discovered in the second portion of the duodenum, occupying approximately one-third of the lumen circumference (Figure 3). Similar to the neck nodule, biopsies of the duodenal mass showed metastatic, poorly differentiated SCC that was strongly positive for p63 and p16 and negative for CK-7, CK-20, CTX-2, TTF-1, and PSA (Figure 1). The patient was not deemed a candidate for chemotherapy due to his poor prognosis and poor functional status and was eventually discharged on home hospice.

## Discussion

HNSCC most commonly metastasizes to the lung, followed by the bone and liver [1-3]. In general, intestinal metastasis from a primary HNSCC is uncommon, with duodenal metastasis being particularly rare. In a 2010 review of reported HNSCC metastasis to the small bowel, only 12 cases were found. Moreover, of those cases, only one case was a metastasis to the duodenum, with the remaining cases illustrating seven ileal metastasis, three jejunal metastasis, and one case being unspecified [6]. Further literature review illustrated two cases of duodenal metastasis, one from Ando et al. (2014) presenting as an intestinal obstruction and the other from Tarangelo et al. (2018) demonstrating simultaneous gastric and duodenal metastasis [7,8]. Additionally, PM from SCC is another rarity in the medical literature. A PubMed search demonstrated five cases of PM from HNSCC [10-13]. PM often results from malignancies of peritoneal organs, including gastric, colon, pancreatic, and ovarian cancer, and are thus frequently adenocarcinomas [15]. Very few cases of PM originating from the SCC of organs such as the esophagus, bladder, and ovaries have been reported [16-18]. To the best of our knowledge, this would be the sixth reported case of PM from HNSCC, and the first reported case involving a simultaneous duodenal metastasis and PM. The independent and combined rarity of HNSCC metastases to the duodenum and peritoneum highlights the extremely unique nature of our case.

Head and neck cancers can arise from several anatomic sites, including the oral cavity, pharynx, and the larynx. Histological analysis and imaging techniques such as CT scan are important for the diagnosis and staging of head and neck tumors. Majority of head and neck cancer cases are SCCs and often develop through a series of transformations from premalignant entities due to carcinogen exposure such as tobacco or alcohol use [1-3]. Tumors are often categorized into three major groups, namely, well differentiated (more than 75% keratinization), moderately differentiated (25% to 75% keratinization), and poorly differentiated tumors. In addition to histological analysis, immunohistochemical staining often helps differentiate different tumors. In general, overexpression of the tumor suppression protein p16 can be seen in SCC and is associated with human papillomavirus infection. Moreover, p63 expression is another protein that is often found in SCCs and helps differentiate from adenocarcinomas [1-3,6]. In this case, both the initial neck nodule biopsy and the GI metastatic lesion were found to have poorly differentiated pathology with immunohistochemical stains positive for p16 and p63.

In the previously documented cases, after the onset of prostrate cancer, a patient’s prognosis was found to be extremely poor, ranging from a few days to a few months [10-12]. In the present case, the patient also demonstrated a poor prognosis and died less than one month after discharge from the hospital to hospice care. In general, PM (including omental carcinomatosis) can rapidly progress and lead to malignant ascites. Chemotherapy and peritoneal excision in certain cases, including gynecological and GI adenocarcinomas, have shown to be viable treatment options. Such procedures have demonstrated an impact on survival and were found to increase the quality of life for patients [19]. Moreover, for duodenal obstruction secondary to metastasis, palliative stenting has been done and reported in the medical literature to improve the quality of life [7]. Such treatment options were considered in the present case; however, due to rapid disease progression and the patient’s poor functional status they were deemed not feasible.

There have been many speculated mechanisms regarding intestinal and PM, such as direct extension of an intraabdominal malignancy, tumor rupture or trauma leading to seeding of the peritoneum, hematogenous dissemination, and even an abnormality in the lymphatic drainage [7,13]. In our case, the patient did not have any abdominal surgeries and had multiple metastatic sites, including the duodenum, making dissemination by hematogenous or lymphatic drainage more likely. In any case, further studies are needed to understand the mechanism involved in HNSCC metastasis.

In general, a high index of suspicion for malignancy should be considered in patients with unexplained anemia. There are currently no screening recommendations that have been proven to increase survival for HNSCC; however, clinicians should be wary of high-risk patients. Guideline-based, age-appropriate cancer screening should be pursued in such patients. For this patient, his history of smoking likely contributed to his SCC as it is a well-known risk factor [1-3]. In hindsight, this patient would have benefited from low-dose CT scan screening for lung cancer, which may have identified early signs of malignancy such as hilar LAD. In the setting of his anemia, this may have prompted a more aggressive workup, which could have affected treatment options and morbidity. Moreover, it is unclear what role earlier endoscopic screening will play in a patient with a history of HNSCC given the fact that it rarely metastasizes to the GI tract. However, this patient would have benefited from a colonoscopy given his age and anemia, and he had alarm symptoms of weight loss, anemia, episodes of dark stool, and persistent vomiting which was an indication for upper endoscopic evaluation. As research ensues, guidelines on HNSCC screening may adapt; however, until then, routine risk stratification and modification, age-appropriate cancer screening, and yearly physical examination should be pursued.

## Conclusions

Here, we described the first case of concurrent duodenal and PM from an HNSCC. Although there has been much speculation as to the mechanism of metastasis from HNSCC to the peritoneum or GI tract, further research is still needed to develop a more comprehensive understanding of the process. As more is learned about head and neck cancers, guidelines on HNSCC screening may change. Nevertheless, clinicians should continue to address risk modifications in high-risk patients, focus on age-appropriate cancer screening, and pursue a thorough yearly physical examination of their patients.
